# Association of Maternal Regulatory Single Nucleotide Polymorphic CD99 Genotype with Preeclampsia in Pregnancies Carrying Male Fetuses in Ethiopian Women

**DOI:** 10.3390/ijms21165837

**Published:** 2020-08-14

**Authors:** Tsehayneh Kelemu, Lena Erlandsson, Daniel Seifu, Markos Abebe, Sisay Teklu, Jill R. Storry, Stefan R. Hansson

**Affiliations:** 1 Department of Biochemistry, College of Health Sciences, Addis Ababa University, P.O. Box 9086 Addis Ababa, Ethiopia; tkelemu_2005@yahoo.com (T.K.); dseifu@ughe.org (D.S.); 2 Department of Obstetrics and Gynecology, Institute of Clinical Sciences Lund, Lund University, 221 85 Lund, Sweden; lena.erlandsson@med.lu.se; 3 Department of Biochemistry, Division of Biomedical Sciences, University of Global Health Equity, P.O. Box 6955 Kigali, Rwanda; 4 Armauer Hanson Research Institute, P.O. Box 1005 Addis Ababa, Ethiopia; markosabebe@yahoo.com; 5 Department of Obstetrics and Gynecology, College of Health Sciences, Addis Ababa University, P.O. Box 9086 Addis Ababa, Ethiopia; siteet@yahoo.com; 6 Department of Hematology and Transfusion Medicine, Division of Laboratory Medicine, Lund University, 221 85 Lund, Sweden; jill.storry@med.lu.se

**Keywords:** SNP, male fetus, preeclampsia, GATA3, CD99

## Abstract

Preeclampsia (PE) is a human specific syndrome with unknown etiology causing maternal and fetal morbidities and mortalities. In PE, maternal inflammatory responses are more exaggerated if the fetus is male than female. Other pregnancy complications such as spontaneous abortions are also more common if the fetus is male. Recent transcriptome findings showed an increased expression of CD99 in erythroid cells from male cord blood in PE. The single nucleotide polymorphism (SNP) rs311103, located in a GATA-binding site in a regulatory region on the X/Y chromosomes, governs a coordinated expression of the Xg blood group members CD99 and Xg^a^ in hematopoietic cells in a sex-dependent fashion. The rs311103C disrupts the GATA-binding site, resulting in decreased CD99 expression. We aimed to investigate the association between PE and the allele frequency of rs311103 in pregnancies in a fetal sex-dependent fashion. In a case-controlled study, we included 241 pregnant women, i.e., 105 PE cases and 136 normotensive controls. A SNP allelic discrimination analysis was performed on DNA from maternal venous blood and fetal cord blood by qPCR. A statistically significant association was observed between rs311103 allele frequency and PE in mothers carrying male fetuses. Therefore, the rs311103 genotype may play a role in the pathogenesis of PE in a fetal sex-specific manner.

## 1. Introduction

The maternal mortality rates in sub-Saharan African countries including Ethiopia (590 maternal deaths per 100,000 live births) remain the highest in the world [1]. Preeclampsia (PE) is the major cause of maternal mortality, accounting for more than 50,000 maternal deaths globally each year, the majority of which occur in developing countries [2]. The syndrome affects 2–8% of pregnancies [3]; it is a human-specific pregnancy complication, with clinical manifestations occurring after 20 weeks of gestation. According to the ISSHP guidelines, maternal manifestations are hypertension in combination with proteinuria and/or other maternal organ dysfunction, including utero-placental dysfunction with fetal growth restriction (FGR) [4]. The etiology is not fully understood, although genetic and environmental pollution factors such as polycyclic aromatic hydrocarbons have been implicated [5,6]. The disease develops in two stages [7]: the first stage is failed spiral artery remodeling that results in uneven blood perfusion and poor placentation, leading to oxidative stress in the placenta; the second stage includes the clinical manifestations, occurring after 20 weeks of gestation. Oxidative stress and tissue damage is suggested to cause a breach in the placenta-barrier, leading to the leakage of fetal and placenta-derived factors into the maternal circulation [8]. This leads to maternal endothelial dysfunction and vasoconstriction, resulting in systemic hypertension, edema and end-organ hypo-perfusion. This process also triggers an immune response, causing inflammation in the mother [8]. Treatment of PE is symptomatic [9], and currently, no cure is available for the condition except for termination of the pregnancy [10]. Despite the benefits of early detection of PE, there is still a lack of specific predictive biomarkers for clinical use to enable early diagnosis. A large effort has been made to define a profile of early biochemical markers in maternal serum to help predict adverse pregnancy outcomes such as PE, and several factors have been proposed on their own or in combinations [11,12]. It has also been suggested that cellular profiles of maternal blood during the first trimester could be indicators of an increased risk of developing PE [13].

Several studies have shown an association between fetal sex and PE. The rate of death of male fetuses in early gestation is higher than that of female fetuses [14]. The obstetric outcomes are determined by complex interactions between the mother, the placenta and the fetus in a fetal sex-dependent fashion [15]; the exact mechanisms, however, are not known. Recent transcriptome findings by Masoumi et al. gave an indication that CD99 expression is upregulated in umbilical cord blood erythroid cells in male fetuses from PE pregnancies [16].

The proteins CD99 and Xg belong to the Xg blood group system. The Xg glycoprotein shows ~48% sequence homology with CD99 [17], however, little is known about the Xg protein and its function. The Xg has been shown to be expressed on erythrocytes and lymphocytes [18]. The CD99 protein is a 32 kDa transmembrane protein encoded by the CD99 gene [19]. The CD99 is expressed at low levels in every cell type, although higher expression is observed in endothelial and hematopoietic cells. CD99 has roles in diverse physiological and pathological processes [20], such as apoptosis [21,22] and cellular differentiation [23,24]. It also serves as a cell surface adhesion molecule for T cells [19]. In addition, CD99 plays a role in the migration of leukocytes to inflamed sites through endothelial junctions and basement membranes of blood vessels [25,26].

The Paired Ig-like type 2 Receptors (PILRs) of CD99 are expressed on immune cells as transmembrane proteins. PILRs are one of the paired receptor families, which are composed of an activating β- and an inhibitory α-isoform. The β isoform is expressed at higher levels on Natural Killer (NK) cells, B cells, T cells, dendritic cells, granulocytes and macrophages, while the α-isoform is expressed at very low levels on NK cells [27]. Takheaw et al. showed that the interaction of a recombinant CD99 with its ligand on immune cells upregulated the production of pro-inflammatory cytokines [28].

The CD99 gene is located within the pseudo-autosomal regions of the X and Y chromosomes [29] and is situated upstream of the XG gene that encodes the Xg^a^ blood group antigen [30]. The single nucleotide polymorphism (SNP) rs311103 is located in a GATA-binding site in a regulatory region between CD99 and XG that co-regulates the expression of the Xg^a^ and CD99 proteins [31]. The minor allele of rs311103 (G > C) interrupts a GATA-binding site, which has been shown [32] to have high affinity for GATA1, GATA2 and GATA3 transcription factors. GATA1 is an erythroid-specific transcription factor and a rs311103 CC genotype completely disrupts the XG transcription in erythroid cells, resulting in the Xg(a-) phenotype. However, erythroid *CD99* expression is not completely controlled by this transcription factor binding site, and CD99 is strongly expressed in individuals of the GG and GC genotypes, while it is low in the CC genotype [33]. While GATA1 is erythroid-specific, GATA2 is an important transcription factor for hematopoietic stem cells and later progenitors that subsequently give rise to lymphocytes, monocytes and neutrophils. GATA3 has been shown to be essential for multi-organ development, and, like the others, regulates tissue-specific-differentiation [34]. GATA3 is broadly expressed in various hematopoietic cells and is important for T cell and NK cell maturation and function [35,36].

Pregnancies with male fetuses have been associated with increased risk for PE, and are believed to be confronted by stronger intrauterine selection forces compared to pregnancies with female fetuses [37,38]. However, the molecular mechanisms behind this have not been fully elucidated. We set out to investigate whether there was an association between maternal SNP rs311103 genotype frequencies and PE in a fetal sex-dependent fashion in an Ethiopian pregnant cohort.

## 2. Results

From a total of 273 participants that were initially recruited, 32 were excluded due to either multiple pregnancies, duplicate codes or lack of fetal sex identity data for the infants. In total, 241 matched maternal and fetal DNA samples were included for genotyping (PE cases, *n* = 105, controls, *n* = 136) (Table 1). In the control group, age was missing for one participant while systolic BP (sBP) and diastolic BP (dBP) were missing for 6 participants. In the PE group, sBP and dBP were missing for 4 participants. A gestational age (GA) of < 34 weeks was considered early-onset PE, while 34 weeks and above was considered to be late-onset PE. Early-onset PE was observed in 23.8% of cases. We calculated the mean values for continuous variables such as age, BP and GA.

We tabulated stepwise frequency distributions of the SNP rs311103 genotypes among mothers and fetuses, and Chi-square was used to test the relationship between genotypes and PE. First, frequency tabulation was done between different maternal groups (Table S1) and among male and female fetuses (Table S2), but no statistically significant association of the SNP rs311103 genotype frequencies between the different groups were found. However, it was noted that PE mothers with the CC genotype carrying males showed a tendency to be lower in number than any other combinations of genotypes.

High CD99 expressing genotypes (GG and GC), designated as G+, and low CD99 expresser genotype (CC) were tabulated against subgroups of mothers. The relationships were tested using the Chi-square test. The frequency distribution of genotypes was compared between PE and control mothers of male fetuses; there was a statistically significant association in the genotype frequencies (*p*-value < 0.05 at X^2^ = 3.94, percentage point 3.84) (Table 2). However, there was no statistically significant association in frequency distribution of the genotypes when comparing PE and control mothers of female fetuses (Table 2).

The frequency distribution of genotypes between male or female fetuses of PE and control mothers was also compared, but no statistically significant associations was found (Table 3).

In addition, the different groups of fetal SNP rs311103 genotypes were tabulated against the mothers (Tables S3 and S4). The distribution of the CC genotype was lower in male fetuses and mothers of PE pregnancies compared to the other groups. 

## 3. Discussion

Our study showed a statistically significant association between the maternal SNP rs311103 genotype frequency and PE pregnancies carrying male fetuses. The maternal SNP rs311103 genotype CC that results in low CD99 expression was underrepresented in PE cases with male fetuses, while there was no difference in frequencies in female pregnancies. There is a lack of evidence regarding a molecular basis for the association between fetal sex and PE. Our previous study showed that CD99 mRNA was upregulated in umbilical cord blood erythroid cells from male fetuses in PE pregnancies [16], and we speculated that CD99 expression, regulated by the SNP rs311103 genotype, might play a role in the pathogenesis of PE in a fetal sex-dependent fashion.

**Figure 1:**
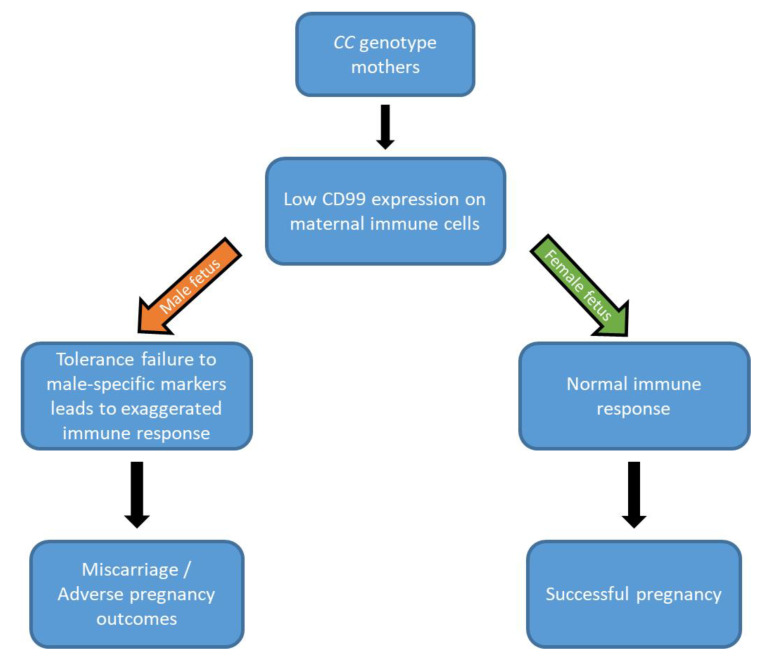
Adverse pregnancy outcomes in male pregnancy. In CC mothers, low CD99 expression may have an effect on pregnancy outcome in a fetal sex-dependent manner. In the case of a male fetus, the maternal immune tolerance is abnormal and the pregnancy outcome is more likely to be adverse, whereas in the case of a female fetus, the maternal immune tolerance is normal, and the pregnancy is more likely to be successful.

Fetal sex is a risk factor for adverse pregnancy outcomes [15]; pregnancies with a male fetus are at increased risk of pregnancy complications such as PE [39], as well as spontaneous abortion and miscarriage [14,38,40]. Fetal mortality rates also vary in a sex-dependent manner; perinatal mortality rates are higher in male compared to female fetuses [38]. Inflammatory responses are features of a normal pregnancy, but are further exaggerated in complicated pregnancies such as PE [41]. Also, the maternal immune response varies in a fetal sex-dependent fashion, where a further increased expression of maternal and placental pro-inflammatory cytokines and decreased levels of immune-regulatory cytokines have been shown in PE pregnancies carrying male fetuses [37,42]. A study suggested that the sex of the offspring may be one of the determining factors for the success of the implantation, where male embryos may be more vulnerable to the process of implantation than females [38]. Our finding is suggestive of implantation failure or failed pregnancy in mothers of genotype CC if the embryo is male (Figure 1).

The SNP rs311103 is situated in a GATA-binding site located in the pseudo-autosomal regions of the sex chromosomes, and has been shown to have high affinity for GATA1, GATA2 and GATA3 transcription factors [32]. The binding of GATA1, GATA2 or GATA3 is functional if the rs311103 is a G, but disrupted if it is a C [31,32], where GG or GC genotypes result in high CD99 expression and the CC genotype in low CD99 expression. GATA3 is an important transcription factor for the maturation and function of immune cells such as the T and NK cells [32,34], and thus, is involved in CD99 expression in these cells. Both T and NK cells play important immunological roles in pregnancy [43]. Type 1 and type 2 T helper (Th1 and Th2) cells play different roles in the outcome of a pregnancy. During a normal pregnancy, there is a shift from a Th1 to a Th2 immune response [44]. The Th2 cells at the feto-maternal interface produce cytokines that promote a successful pregnancy by inhibiting the Th1 response, while Th1 cells produce cytokines that are associated with pregnancy complications such as recurrent pregnancy losses and implantation failure [43,44,45]. During early pregnancy, the uterine NK (uNK) cells become the predominant leukocyte population in the maternal uterine decidua and interact with embryonic trophoblast cells at the implantation site [46]. The uNK cells secrete cytokines that play a role in the trophoblast invasion and spiral artery remodeling to establish the nutrient supply to the developing fetus and placenta [47]. Failure in the spiral artery remodeling results in poor placentation in stage 1 of PE, which leads to increased oxidative stress in the placenta and, eventually, to the clinical manifestations seen in stage 2 [48]. CD99 is also expressed in endothelial cells and is important for the diapedesis of leukocytes through the blood vessel wall [25], and we suggest it could be important for recruitment of NK and T cells to the uterine decidua.

The GATA3 transcription factor binding to the GATA-binding site would regulate the CD99 expression in both maternal T and NK cells present in the uterine decidua, and the maternal CC genotype would then result in low levels of CD99 expression and protein on these cells. Since both T and NK cells are important for a normal placentation, a low CD99 expression on these cells might have negative effects on the process, and may contribute to a failed implantation. Maternal low CD99 levels in immune cells such as Th cells might disturb the balance between Th1 and Th2 responses, and thereby, further increase the maternal inflammatory response seen during PE pregnancies. Low levels of CD99 on endothelial cells in maternal blood vessels would have a negative effect on the recruitment of leukocytes to the uterus, and further disturb the balance between the important immune cells needed during placentation. Furthermore, it is possible that low levels of CD99 on the endothelial cells lining the spiral arteries might affect the remodeling process and contribute to poor placentation. However, our results suggest that low levels of maternal CD99 are only important when the embryo is male. Our recent study showed an association between PE and a maternal killer cell immunoglobulin-like receptor (KIR) inhibitory genotype expressed in the uNK cells, demonstrating the important role played by uNK cells in pregnancy outcome [49]. However, there was no association between the maternal rs311103 genotype frequencies described in this study and the maternal KIR genotypes previously described. The challenge of finding suitable biomarkers for early clinical diagnosis of PE remains, and future studies will show if the molecular mechanisms that link CD99 expression in PE pregnancies with male fetuses have any potential as part of a biomarker panel.

A weakness of our study is the incomplete collection of data during recruitment, resulting in a reduction of the number of samples included in the final analyses.

In conclusion, our study suggests that mothers with the CC genotype developing PE have an increased rate of implantation failure or failed pregnancy if the embryo is male in a fetal sex-dependent fashion. The molecular mechanisms that link CD99 expression in PE pregnancies with male fetuses is currently lacking, but our data may be helpful in directing future studies towards investigations of exaggerated sex-dependent maternal immune responses towards male fetuses.

## 4. Materials and Methods

### 4.1. Study Design and Study Participants

A case-control study design was employed. Study participants were recruited at Adama Regional Referral Hospital, Ethiopia. Data collection was performed by midwife nurses and laboratory staff who received training about data collection. The principal investigator and head nurse supervised the data collection process. A standard operating procedure was prepared and followed for data collection. A national protocol was used by the Obstetrics and Gynecology specialist physicians and medical residents for the identification of PE cases during their routine hospital practice (Management Protocol on Selected Obstetrics Topics, Federal Democratic Republic of Ethiopia, Ministry of Health, 2010). Participant recruitment, the recording of clinical and demographic data and blood sample collections took place from December 2016 to August 2017. A total of 241 pregnant women, of which 105 were PE cases and 136 normotensive controls, were included. The participants were recruited based on the order of their visit to the Obstetrics and Gynecology delivery ward. The cohort has been used in a previous study [49].

### 4.2. Ethical Considerations

Ethical clearance was secured from Department of Biochemistry, School of Medicine, College of Health Sciences, Addis Ababa University, Ethiopia (Protocol number 013/16/Biochem Form AAUMF 03-008, 20 May 2016 and 8 May 2018); AHRI-ALERT Ethical Review Committee (Project Reg. No. P015/16; 2 August 2016–1 August 2017); NRERC, the Federal Democratic Republic of Ethiopia Ministry of Science and Technology (Date: 13/2017 Ref. No. 3.10/186/2018, 3/0/86/2018, Date: 14/05/2018. All subjects gave written informed consent for participation in the study. The study was conducted in accordance with the Declaration of Helsinki.

### 4.3. Inclusion Criteria

We included pregnant women that were 18 years and above in both PE and control groups. In the PE group, we included pregnant women with gestational hypertension (two BP readings of dBP ≥90 mmHg and/or sBP ≥140 mmHg four hours apart) at or after 20 weeks of gestation with proteinuria (spot urine protein ≥ 2+ on dipstick testing or ≥30 mg/mg protein/creatinine) or findings of maternal organ dysfunction or utero-placental dysfunction (fetal growth restriction) [50]. In the control group, we included normotensive healthy singleton pregnancies, with no clinical signs or laboratory findings of organ dysfunction or utero-placental dysfunction.

### 4.4. DNA Extraction and Genotyping

The maternal venous blood and cord blood samples were collected in EDTA test tubes. The samples were stored at –20 °C until extractions of genomic DNA were performed using QIAGEN DNA Mini Blood Kit (QIAGEN, Hilden, Germany) from 200 μL of maternal blood or 200 μL of cord blood. The DNA was used for SNP rs311103 genotyping based on the primers and methods previously described [31] using TaqMan^TM^ Genotyping master mix (Applied Biosystems by Thermo Fisher Scientific, Waltham, MA, USA). Briefly, the maternal and fetal genomic DNA was amplified in a 10-µL, real time quantitative polymerase chain reaction containing 1 µL of 10 ng genomic DNA, 5 µL of 2× TaqMan Genotyping master mix, 0.25 µL of 40× primer assay and 3.75 µL of distilled water. Cycling was carried out in a QuantStudio 3 thermocycler (Applied Biosystems by Thermo Fisher Scientific). Positive controls for the three genotypes, i.e., GG, GC and CC, and a negative control (DNase-free water) were included in each assay, together with test samples. All samples were run in duplicate.

### 4.5. Data Analysis

Demographic and clinical data were collected using the COLLECT database developed by the Global Pregnancy Collaboration (CoLab; https://pregnancycolab.tghn.org/collect/). The statistical analysis was done using IBM SPSS version 26.0. The data was analyzed using the Chi-square test. *p*-value < 0.05, at 95% confidence interval, was considered statistically significant.

## Figures and Tables

**Table 1:** Demographic and clinical characteristics of study participants.

Characteristics	Controls (*n* = 136)	PE Cases (*n* = 105)
Age	25.3 ± 5	26.6 ± 5
sBP	114.0 ± 9	149.9 ± 17.2
dBP	72.4 ± 8.1	97.6 ± 10.1
GA	38 + 5 weeks	35 + 6 weeks
Early-onset PE Late-onset PE	n.a n.a	23.8% (25) 76.2% (80)

Data is presented as Mean ± SD. GA: gestational age; PE: preeclampsia; sBP: systolic blood pressure; dBP: diastolic blood pressure; n.a: not applicable.

**Table 2:** Frequency distribution of High and Low CD99 expresser genotypes among subgroups of mothers.

Maternal Genotype	PE Mothers of Males % (*n* = 51)	Control Mothers of Males % (*n* = 77)	PE Mothers of Females % (*n* = 54)	Control Mothers of Females % (*n* = 59)
G+	92.2 (47)	79.2 (61)	77.8 (42)	81.4 (48)
CC	7.8 (4)	20.8 (16)	22.2 (12)	18.6 (11)

PE—preeclampsia.

**Table 3:** Frequency distribution of High and Low CD99 expresser genotypes among subgroups of fetuses.

Fetal Genotype	Males of PE Mothers % (*n* = 51)	Males of Control Mothers % (*n* = 77)	Females of PE Mothers % (*n* = 54)	Females of Control Mothers % (*n* = 59)
G+	80.4 (41)	76.6 (59)	75.9 (41)	79.7 (47)
CC	19.6 (10)	23.4 (18)	24.1 (13)	20.3 (12)

PE—preeclampsia.

## Data Availability

Materials, data and associated protocols are available upon request.

## Supplementary Materials

The following are available online at https://www.mdpi.com/1422-0067/21/16/5837/s1, Table S1: Frequency distribution of SNP rs311103 genotypes among maternal subgroups; Table S2: Frequency distribution of SNP sr311103 genotypes among fetal subgroups; Table S3: Male fetus genotype versus mother genotype; Table S4: Female fetus genotype versus mother genotype.

## Abbreviations

PE	preeclampsia
GA	gestational age
sBP	systolic blood pressure
dBP	diastolic blood pressure
SNP	single nucleotide polymorphism
uNK	uterine natural killer cells
Th	T helper cells
