# Supplementary material for: Association of Maternal Regulatory Single Nucleotide Polymorphic CD99 Genotype with Preeclampsia in Pregnancies Carrying Male Fetuses in Ethiopian Women
Source: Int J Mol Sci. 2020 Aug 14;21(16):5837. doi: 10.3390/ijms21165837 (PMC7461595; doi:10.3390/ijms21165837)
Supplement: Supplementary file 1 [file ijms-21-05837-s001.pdf]

<u>Supplementary information:</u>

Association of maternal regulatory single nucleotide polymorphic CD99 genotype with preeclampsia in pregnancies carrying male fetuses in Ethiopian women

Tsehayneh Kelemu[a], Lena Erlandsson[b], Daniel Seifu[a,c], Markos Abebe[d], Sisay Teklu[e], Jill R. Storry[f] and Stefan R. Hansson[b]*

Table 1. Frequency distribution of SNP rs311103 genotypes among maternal subgroups.

	**Maternal groups**			
**Maternal Genotype**	**PE mothers of males % (n=51)**	**Control mothers of males % (n=77)**	**PE mothers of females % (n=54)**	**Control mothers of females % (n=59)**
GG	41.2 (21)	36.4 (28)	40.8 (22)	40.7 (24)
GC	51.0 (26)	42.8 (33)	37.0 (20)	40.7 (24)
CC	7.8 (4)	20.8 (16)	22.2 (12)	18.6 (11)

PE-preeclampsia

Table S2. Frequency distribution of SNP sr311103 genotypes among fetal subgroups.

**Fetal genotype**	**Males of PE mothers % (n=51)**	**Males of control mothers % (n=77)**	**Females of PE mothers % (n=54)**	**Females of control mothers % (n=59)**
GG	31.4 (16)	29.9 (23)	40.8 (22)	30.5 (18)
GC	49.0 (25)	46.7 (36)	37.0 (20)	49.2 (29)
CC	19.6 (10)	23.4 (18)	22.2 (12)	20.3 (12)

PE-preeclampsia

Table S3. Male fetus genotype versus mother genotype.

	**PE mother genotype**			**Control mother genotype**		
**Male genotype**	**CC**	**GC**	**GG**	CC	GC	GG
CC	3	7	0	11	7	0
GC	1	13	11	5	19	12
GG	0	6	10	0	7	16

PE-preeclampsia

Table S4. Female fetus genotype versus mother genotype.

	**PE mother genotype**			**Control mother genotype**		
**Female genotype**	**CC**	**GC**	**GG**	CC	GC	GG
CC	8	4	0	5	7	0
GC	4	7	9	6	13	10
GG	0	9	13	0	4	14

PE-preeclampsia
